# Stimulant safe supply: a potential opportunity to respond to the overdose epidemic

**DOI:** 10.1186/s12954-019-0351-1

**Published:** 2020-01-10

**Authors:** Taylor Fleming, Allison Barker, Andrew Ivsins, Sheila Vakharia, Ryan McNeil

**Affiliations:** 1 British Columbia Centre on Substance Use, 400-1045 Howe Street, Vancouver, BC V6Z 2A9 Canada; 2 Interdisciplinary Studies Graduate Program, University of British Columbia, 270-2357 Main Mall, Vancouver, BC V6T 1Z4 Canada; 3 Department of Medicine, University of British Columbia, St. Paul’s Hospital, 608-1081 Burrard Street, Vancouver, BC V6Z 1Y6 Canada; 4 Drug Policy Alliance, 131 West 33rd Street, 15th Floor, New York, NY 10001 USA; 5 Department of Medicine, Yale School of Medicine, 367 Cedar Street, New Haven, CT 10001 USA; 6 Program in Addiction Medicine, Yale School of Medicine, 367 Cedar Street, New Haven, CT 10001 USA

**Keywords:** People who use drugs, Stimulant use, Overdose, Drug treatment

## Abstract

**Background:**

Occurring against the backdrop of an overdose crisis, stimulant use and stimulant-involved deaths in North America are increasing at an alarming rate. Many of these deaths are being attributed to fentanyl and related analogs, which have been increasingly found within street-level stimulant supplies. Within this, people experiencing socio-economic marginalization are at the greatest risk of overdose and other harms from adulterated stimulants. Current treatments for stimulant use disorder have limited effectiveness, and even less applicability to the lived realities of marginalized stimulant users. Emerging technologies, such as drug checking, are being implemented to support safer stimulant use, but the accessibility and utility of these technologies to stimulant users are framed by experiences of vulnerability that render them largely ineffective.

**Stimulant safe supply:**

Solutions that provide a legal and safe supply of non-adulterated stimulants of known quality, and within a health care framework, are needed to directly address the risk of an increasingly adulterated stimulant supply. Similar innovative opioid-focused interventions are being piloted with medications that have a similar pharmacological effect as their illicit counterparts. While there are currently no approved pharmacotherapies for stimulant use, research has demonstrated a number of stimulant medications that are promising substitutes for cocaine and methamphetamine use. Much like with opioid-focused pharmacotherapies, having a consistent and safe supply of stimulants can lead to improved health outcomes and will drastically reduce overdose risk. However, for a stimulant safe supply intervention to be a success, it must provide the high and performance-enhancing effects that people seek from the illicit market, which requires doses and user agency that trials to date have not provided.

**Conclusion:**

Efforts are needed to investigate the feasibility of pharmacological stimulant-based interventions that address safe supply needs. The promise of similar opioid-focused approaches in addressing both overdose-related risks and experiences related to vulnerability underscores the need to advance safe supply approaches targeted towards people who use stimulants. Given the current overdose crisis and rising stimulant use across North America, the implementation and evaluation of such novel stimulant-focused interventions should be a public health priority.

## Background

Illicit stimulant use has been steadily rising globally, with reports estimating that more than twice as many people use stimulants than opioids [1]. North America has the highest rates of stimulant use in the world and stimulant-involved deaths are increasing [2]. A growing number of these deaths also involve opioids, possibly as the result of polysubstance use or adulteration of the illicit drug supplies [1, 3]. This is occurring against the backdrop of North America’s overdose crisis, in which the dramatic rise in overdose deaths can primarily be attributed to increases in distribution and use of illicitly manufactured fentanyl, fentanyl-adulterated drugs, and related analogs [4, 5]. Innovative solutions that acknowledge and address the risks of a contaminated drug supply are needed to ensure the health and wellbeing of people who use drugs, including illicit stimulant users.

The United States Centers for Disease Control (CDC) reported a 52% increase in overdose deaths involving cocaine, and a 33% increase in deaths involving other psychostimulants from 2015 to 2016, with surveillance data suggesting synthetic opioid involvement is the main driver of this increase in mortality [3, 4]. Provisional CDC data already shows that nationally between November 2017 and November 2018, while fentanyl involvement remains high, the number of stimulant-involved overdose deaths has grown to be comparable to the number of opioid-involved overdose deaths [6]. A focus on state-specific trends shows even more stark contrasts, including settings where stimulant-involved deaths have even surpassed some opioids. States along the west coast such as Oregon and Nevada are experiencing a methamphetamine-driven overdose crisis while those along the east coast in places like Washington, DC and Maine are seeing increasing rates of cocaine-involved deaths [6]. US Drug Enforcement Agency drug seizure and surveillance data from 2017 suggest that increasing numbers of cocaine and methamphetamine samples tested positive for fentanyl [7]. While these numbers remain low, they have increased in recent years and must be monitored.

Similar Canadian data is limited. However, in British Columbia (BC), Canada, cocaine has been found in 50% of illicit drug overdose deaths, and methamphetamine in 37% of deaths [5], and of these illicit fentanyl was detected in 72% and 77% of cocaine- and methamphetamine-related deaths, respectively [8]. Notably, illicit fentanyl was implicated in 87% of overdose deaths in BC in 2018 [9], suggesting that fentanyl-adulteration is of urgent concern for people who use both opioids and stimulants, particularly those engaged in polysubstance use. Further, emerging drug-checking technologies (DCT) have confirmed that street-level illicit stimulant supplies are being adulterated with fentanyl and other substances (e.g., ground pumice stone, plaster) [10]. Within this, people who use stimulants, both those who are opioid-naive and those who engage in polysubstance use, are at increased risk of overdose death due to the potency of fentanyl and related analogs.

Alongside these growing concerns, there is increased awareness that structurally oppressed people who use drugs experience a disproportionate burden of drug-related harms, including overdose [11]. These poor outcomes can be attributed to the ways in which their location within social hierarchies limits their agency and perpetuates vulnerability to risk and harm [11]. When viewed through a ‘structural vulnerability’ lens, people who use illicit stimulants are understood to be disproportionately impacted by intersecting oppressions (e.g., extreme poverty, housing instability, drug prohibition, and policing). These oppressions are still further shaped by racism, (trans)misogyny, homophobia, and ableism, resulting in disparities in risk and harm among, for example, people of color, women, and gender diverse persons. This is illustrated by research showing that African-Americans consistently have higher rates of cocaine-involved overdose deaths, which are under-investigated and shaped by criminalization [12]. Further, women involved in sex work who use crystal methamphetamine may experience increased vulnerabilities through their relationships with sexual partners (e.g., relying on sexual partners to obtain drugs) [13]. It is, therefore, necessary to be responsive to the structural vulnerabilities of impacted groups during the planning and implementation of any and all interventions seeking to address overdose, including through the meaningful involvement of people who use stimulants.

## Limitations to current interventions for stimulant use

Almost 90% of individuals who meet the criteria for any substance use disorder in the USA have not received substance use treatment in the past year, and this is also true for those with stimulant use disorders [14]. In a systematic review of barriers to methamphetamine treatment access, people who use amphetamines were found to have: low confidence in available treatment options; concerns regarding relevancy and effectiveness of treatment services for methamphetamine use, especially those not tailored to people using stimulants and that include people using opioids; and belief that treatment was unnecessary [15]. Similar attitudes towards treatment have been reported among people using crack cocaine [16]. Moreover, past research has suggested coercive practices in treatment initiation with structurally vulnerable people who use drugs, leading to negative views of treatment and high rates of treatment discontinuation [17]. Some of this may be due to the fact that no “gold-standard” treatments exist for cocaine or methamphetamine use disorders [18]. In addition, the implementation and availability of promising or evidence-based interventions remains inconsistent across North America and globally. It also must be noted that the needs of the highest-risk people (e.g., individuals experiencing homelessness) who use stimulants often cannot be met simply through psychosocial or medical treatments alone—many need comprehensive social, psychiatric, and economic supports as well.

Unlike opioid use, which is discussed in the current discourse as a medical issue to be pharmacologically treated, traditional treatment of stimulant use disorders generally involves psychosocial interventions such as twelve-step facilitation (TSF), cognitive behavioral therapy (CBT), and contingency management (CM). TSF is among the most common psychosocial approachs used in North America today [19], grounded in the philosophies of Alcoholics Anonymous and Narcotics Anonymous and framing substance use disorder as a life-long disease requiring sustained abstinence. According to this philosophy, recovery can be maintained through regular attendance in self-help support groups and committing to personal and spiritual growth by advancing through its prescribed 12-step program. TSF as a clinical approach in substance use treatment settings is meant to prime clients into the philosophy and to facilitate involvement in the self-help groups as facilitators of long-term recovery. However, TSF has been fraught with controversy about its spiritual framing, and evidence indicates that it has limited effectiveness [1, 20]. In brief, CBT aims to modify patterns of thinking that are believed to lead to drug use, and to support patients in developing healthier coping mechanisms [21]. CM also seeks to change substance use-related behavioral patterns by providing a reward (e.g., cash, gift cards) contingent upon the patient providing a urine sample to prove they have been abstinent from stimulants [20]. However, evidence supporting the efficacy of these approaches also raises questions about their long-term sustainability, as their effects often diminish, or disappear entirely, once the intervention is discontinued [1, 20].

Psychosocial interventions can also serve to reinforce social norms that frame stimulant use solely as a ‘behavioral problem’ and which overlooks evidence that stimulant use is largely driven by structural and environmental factors, including instrumental uses of stimulants. For some people who use stimulants, particularly those reliant upon methamphetamine’s performance-enhancing effects (e.g. increased wakefulness, productivity, focus), promoting abstinence does not address instrumental uses of stimulants or underlying conditions that frame stimulant use. Promoting abstinence also minimizes the very real role that satisfaction and pleasure can play in sustaining stimulant use and that may not immediately be met by pursuing abstinence. Given this, interventions to address stimulant use should shift beyond ‘treating’ stimulant use disorder by promoting reduced or abstinent stimulant use to supporting provision of a safe supply of stimulants, especially within the context of an increasingly adulterated illicit stimulant supply.

## Supporting safer stimulant use through a safe supply

While interventions, such as DCTs, are progressively being promoted as strategies to support safer stimulant use, these often fail to account for the lived realities of those most vulnerable to drug-related risks (e.g., overdose). Recent studies have found acceptability of DCTs to be high among people who use drugs [22, 23], but also that these are of low priority within the context of an established dealer-client relationship [24]. Further, those among the most marginalized members of society (e.g., homeless and racialized individuals) have expressed a low likelihood of actually utilizing these services, as they are unable to easily replace drugs found to be adulterated due to extreme poverty [22], and adulteration is becoming an expectation of the street-level drug supply [23]. DCTs are also often implemented in supervised consumption sites (SCS), which themselves serve as overdose prevention interventions. Within North America’s limited number of SCSs, these have largely focused on injecting and only one regulated supervised inhalation site exists [25], rendering people who smoke stimulants vulnerable to overdose and other harms associated with unsupervised drug use.

Solutions that seek to minimize, or eliminate altogether, the risks of an adulterated drug supply through the provision of a safe supply (i.e., legal, non-adulterated, of known quality, and with user agency in consumption practices) of stimulants are urgently needed as part of a more comprehensive response to the overdose crisis. There has been recent debate regarding the need for a legal and safe supply for opioids, administered as a part of health and social care. Multiple studies have also piloted innovative injectable diacetylmorphine and hydromorphone [26, 27] as medications for addressing the harms of opioid use disorder and providing alternatives to illicit drugs. The promise of these approaches in addressing both overdose-related risks and experiences related to structural vulnerability (e.g., illicit income generation) points to the need to advance similar approaches to address the risk of overdose and other harms among people who use stimulants, particularly within the context an adulterated stimulant supply.

The United Nations Office on Drugs and Crime’s (UNODC) recent report on treatment of stimulant use disorders summarizes emerging evidence demonstrating the treatment potential of prescription psychostimulant substitution and provides recommendations for a pharmacological treatment model that is instructive for stimulant safe supply approaches [1]. The report discusses that, for example, the psychostimulant methylphenidate may be an appropriate treatment for methamphetamine use disorder [28] but not for cocaine use disorder [29]; whereas high doses of extended-release amphetamines have been found to be useful for addressing cocaine dependence [30]. Other psychostimulants that have been considered and reported on in recently published systematic reviews include modafinil, bupropion, dexamphetamine, and mazindol [31, 32]. Notably, there are currently no approved pharmacotherapies for treatment of stimulant use disorder and any practical uses of these medications would be considered “off-label.” Methylphenidate, extended-release amphetamines, and other candidate medications for use as a safe supply, which all can be taken orally every day, have potential because they have a similar pharmacological effect to their illicit counterparts [1]. In treatment-oriented trials these stimulant substitutes were administered in dosages and formulations (e.g., extended-release) that provided a consistent level of stimulation throughout the day, as opposed to a single large, rapidly acting dose that produces a “high.” Given that many stimulant users desire the “high,” any proposals for stimulant safe supply would need to acknowledge and emphasize user agency in diverse consumption practices.

Access to a consistent supply of stimulants of known quality can possibly lead to the same improved health outcomes observed among participants in injectable hydromorphone and diacetylmorphine interventions, such as reductions in abscesses [33], transmission of infectious disease (e.g., hepatitis C, HIV) [34], early mortality [35], and reduced engagement with law enforcement [36]. Additionally, stimulant safe supply approaches may provide opportunities to engage hard-to-reach populations in care in situations that are not informed by crisis (e.g., hospitalization, overdose). Ongoing engagement in safe supply programming could also allow for medical staff to monitor and care for participants whose use could be associated with risk for coronary disease, low body weight, anxiety, psychosis, and other health concerns [37]. Through therapeutic and nonjudgmental service provision, providers may also be able to facilitate access to other forms of treatment, although the harm reduction goals (e.g., overdose prevention) should be prioritized.

Drug user activist groups have recently argued the necessity of a safe stimulant supply in Canada, acknowledging that any form of medication-based approach is likely to fail if it does not mirror what people who use drugs seek (i.e., the “high”) in the illicit market [38]. Current candidate medications have thus far only been studied in the context of treatment of stimulant use disorder, with the eventual goal of reductions in, or abstinence from, stimulant use. To date, trials have not included doses that would enable participants to achieve the “high” associated with a medication’s illicit proxy or to freely use these medications (e.g., user agency in dose amount, frequency, method of consumption). Thus, to study current candidate medications in a safe supply context will require that medications be administered in potentially larger doses than have previously been investigated, and potentially more frequently than once per day. Efforts are urgently needed to investigate the feasibility of pharmacological stimulant-based interventions that address safe supply needs and the underlying risk that a fentanyl-contaminated supply poses to people who use stimulants or are polysubstance users, as well as the lack of non-abstinence-based treatment options.

Meaningfully involving people who use stimulants in intervention planning and implementation will be further important in acknowledging their lived realities. Models that include people with lived experience as partners in their own care exist and have demonstrated success in implementing programs and interventions that are more responsive and relevant which, importantly, have a high likelihood of uptake within their intended communities [39, 40]. This is also a necessary process to design a stimulant safe supply intervention that is low threshold, and thus, accessible to structurally vulnerable people who use stimulants. These populations, who are often considered to be “hard to reach” due to stigma and systematic marginalization, experience challenges in accessing traditional health care, let alone innovative harm reduction interventions. Research on low-threshold programs involving the use of injectable hydromorphone and diacetylmorphine has characterized the primary goal as harm reduction, rather than abstinence or reduced drug use, and emphasized that participation in such interventions must not be punitively impacted by ongoing illicit drug use [41]. Questions surrounding low-threshold delivery for prospective stimulant safe supply interventions should be guided by participatory planning so as not to reproduce operating procedures (e.g., attendance policies, urine drug testing) that create barriers to access and engagement.

## Conclusions

There is an urgent need to pursue stimulant safe supply approaches and the importance of meaningfully involving people who use stimulants in the planning of these interventions cannot be overstated. Within the context of drug criminalization, the risk of adulteration is inherent to the illicit drug market, which with continued prohibition has the potential to become increasingly more lethal. While all people using stimulants are at risk, the risk is especially pronounced among socially and economically marginalized individuals for whom multiple intersecting oppressions limit agency and produce vulnerability to risk and harm. Extending safe supply interventions to include stimulants would provide opportunities to address overdose risk and drug-related harms in ways current models of psychosocial treatment are unable. Possible models that could inform the implementation of pilot stimulant safe supply interventions exist, including low-threshold injectable hydromorphone and diacetylmorphine interventions, and could be adapted to meet the needs of people who use stimulants through participatory planning processes. Such a move would signal that illicit stimulant use and associated health impacts indeed matter. Importantly, the ways in which stimulant use is differentiated from opioid use in the current discourse, with the latter more widely accepted as a medical concern and treated with medication-based therapies, risks undermining efforts to make more treatment options (e.g., pharmacotherapies) available. Given the current overdose epidemic in North America, and rising stimulant and polysubstance use, the implementation and evaluation of novel stimulant-focused interventions are of critical importance.

## References

[1] United Nations Office on Drugs and Crime (2019). Treatment of stimulant use disorders: current practices and promising perspectives.

[2] United Nations Office on Drugs and Crime. World drug report 2018. Vienna, AT: United Nations publication; June 2018.

[3] Kariisa MSL, Wilson N, Seth P, Hoots B (2019). Drug overdose deaths involving cocaine and psychostimulants with abuse potential — United States, 2003–2017. MMWR Morb Mortal Wkly Rep.

[4] Centres for Disease Control and Prevention. Overdose deaths involving opioids, cocaine, and psychostimulants — United States, 2015–2016 2018 [Available from: https://www.cdc.gov/mmwr/volumes/67/wr/mm6712a1.htm#T1_down. Accessed 24 May 2019. 10.15585/mmwr.mm6712a1 PMC5877356 29596405

[5] BC Coroners Service. Fentanyl-detected illicit drug overdose deaths: January 1, 2012 to March 31, 2019. BC Coroners Service; 2019.

[6] Ahmad FB, Escobedo LA, Rossen LM, Spencer MR, Warner M, Sutton P. Provisional drug overdose death counts National Center for Health Statistics: National Center for Health Statistics; 2019 [Available from: https://www.cdc.gov/nchs/nvss/vsrr/drug-overdose-data.htm. Accessed 23 June 2019.

[7] United States Drug Enforcement Administration. 2018 National drug threat assessment. October 2018.

[8] Ministry of Public Safety and Solicitor General. Illicit drug overdose deaths in BC: findings of coroners’ investigations. BC Coroners Service; 2018.

[9] BC Coroners Service. Illicit drug overdose deaths in BC January 1, 2008 - March 31, 2019. BC Coroners Service; 2019.

[10] Tupper KW, McCrae K, Garber I, Lysyshyn M, Wood E (2018). Initial results of a drug checking pilot program to detect fentanyl adulteration in a Canadian setting. Drug Alcohol Depend.

[11] Rhodes T, Wagner K, Strathdee SA, Shannon K, Davidson P, Bourgois P, O’Campo P, Dunn JR (2012). Structural violence and structural vulnerability within the risk environment: theoretical and methodological perspectives for a social epidemiology of HIV risk among injection drug users and sex workers. Rethinking social epidemiology: towards a science of change.

[12] Seth P, Scholl L, Rudd RA, Bacon S (2018). Overdose deaths involving opioids, cocaine, and psychostimulants — United States, 2015-2016. Morb Mortal Wkly Rep.

[13] Shannon K, Strathdee S, Shoveller J, Zhang R, Montaner J, Tyndall M (2011). Crystal methamphetamine use among female street-based sex workers: moving beyond individual-focused interventions. Drug and Alcohol Dependence..

[14] Substance Abuse and Mental Health Services Administration. Key substance use and mental health indicators in the United States: results from the 2017 National Survey on Drug Use and Health. Rockville, MD: Center for Behavioral Health Statistics and Quality, Substance Abuse and Mental Health Services Administration; 2018.

[15] Cumming C, Troeung L, Young JT, Kelty E, Preen DB (2016). Barriers to accessing methamphetamine treatment: a systematic review and meta-analysis. Drug Alcohol Depend.

[16] Wechsberg WM, Zule WA, Riehman KS, Luseno WK, Lam WK (2007). African-American crack abusers and drug treatment initiation: barriers and effects of a pretreatment intervention. Subst Abuse Treat Prev Policy.

[17] Damon W, Small W, Anderson S, Maher L, Wood E, Kerr T (2017). 'Crisis' and 'everyday' initiators: a qualitative study of coercion and agency in the context of methadone maintenance treatment initiation. Drug Alcohol Rev.

[18] Fischer B, Blanken P, Da Silveira D, Gallassi A, Goldner EM, Rehm J (2015). Effectiveness of secondary prevention and treatment interventions for crack-cocaine abuse: a comprehensive narrative overview of English-language studies. Int J Drug Policy.

[19] Substance Abuse and Mental Health Services Administration. Data on substance abuse treatment facilities. Rockville, MD: Substance Abuse and Mental Health Services Administration; 2018.

[20] Benishek LA, Dugosh KL, Kirby KC, Matejkowski J, Clements NT, Seymour BL (2014). Prize-based contingency management for the treatment of substance abusers: a meta-analysis. Addiction.

[21] Harada T, Tsutomi H, Mori R, Wilson DB. Cognitive-behavioural treatment for amphetamine-type stimulants (ATS)-use disorders. Cochrane Database of Systematic Reviews. 2018(12). 10.1002/14651858.CD011315.pub2 PMC6516990 30577083

[22] Sherman SG, Morales KB, Park JN, McKenzie M, Marshall BDL, Green TC (2019). Acceptability of implementing community-based drug checking services for people who use drugs in three United States cities: Baltimore, Boston and Providence. Int J Drug Policy.

[23] Kennedy MC, Scheim A, Rachlis B, Mitra S, Bardwell G, Rourke S (2018). Willingness to use drug checking within future supervised injection services among people who inject drugs in a mid-sized Canadian city. Drug Alcohol Depend.

[24] Bardwell G, Boyd J, Arredondo J, McNeil R, Kerr T (2019). Trusting the source: the potential role of drug dealers in reducing drug-related harms via drug checking. Drug Alcohol Depend.

[25] Bourque S, Pijl EM, Mason E, Manning J, Motz T (2019). Supervised inhalation is an important part of supervised consumption services. Can J Public Health.

[26] Oviedo-Joekes E, Palis H, Guh D, Marchand K, Brissette S, Harrison S (2019). Treatment with injectable hydromorphone: Comparing retention in double blind and open label treatment periods. J Subst Abuse Treat.

[27] Fairgrieve C, Fairbairn N, Samet JH, Nolan S (2018). Nontraditional alcohol and opioid agonist treatment interventions. Med Clin North Am.

[28] Rezaei F, Emami M, Zahed S, Morabbi M-J, Farahzadi M, Akhondzadeh S (2015). Sustained-release methylphenidate in methamphetamine dependence treatment: a double-blind and placebo-controlled trial. DARU.

[29] Dürsteler-MacFarland KM, Farronato NS, Strasser J, Boss J, Kuntze MF, Petitjean SA (2013). A randomized, controlled, pilot trial of methylphenidate and cognitive-behavioral group therapy for cocaine dependence in heroin prescription. J Clin Psychopharmacol.

[30] Levin FR, Mariani JJ, Specker S, Mooney M, Mahony A, Brooks DJ (2015). Extended-release mixed amphetamine salts vs placebo for comorbid adult attention-deficit/hyperactivity disorder and cocaine use disorder: a randomized clinical trial extended-release mixed amphetamine salts for adhd/cocaine use disorderextended-release mixed amphetamine salts for adhd/cocaine use disorder. JAMA Psychiatry.

[31] Pérez-Mañá C, Castells X, Torrens M, Capellà D, Farre M. Efficacy of psychostimulant drugs for amphetamine abuse or dependence. Cochrane Database Syst Rev. 2013;9. 10.1002/14651858.CD009695.pub2 PMC11521360 23996457

[32] Castells X, Cunill R, Pérez-Mañá C, Vidal X, Capellà D. Psychostimulant drugs for cocaine dependence. Cochrane Database Syst Rev. 2016;9. 10.1002/14651858.CD007380.pub4 PMC6457633 27670244

[33] Armstrong G, Kermode M, Sharma C, Langkham B, Crofts N (2010). Opioid substitution therapy in manipur and nagaland, north-east india: operational research in action. Harm Reduction J.

[34] MacArthur GJ, Minozzi S, Martin N, Vickerman P, Deren S, Bruneau J (2012). Opiate substitution treatment and HIV transmission in people who inject drugs: systematic review and meta-analysis. BMJ.

[35] Sordo L, Barrio G, Bravo MJ, Indave BI, Degenhardt L, Wiessing L (2017). Mortality risk during and after opioid substitution treatment: systematic review and meta-analysis of cohort studies. BMJ.

[36] Oviedo-Joekes E, Guh D, Brissette S, Marchand K, MacDonald S, Lock K (2016). Hydromorphone compared with diacetylmorphine for long-term opioid dependence: a randomized clinical trial hydromorphone vs diacetylmorphine for long-term opioid dependence. JAMA Psychiatry.

[37] Darke S, Kaye S, Duflou J (2017). Rates, characteristics and circumstances of methamphetamine-related death in Australia: a national 7-year study. Addiction.

[38] Canadian Association of People who Use Drugs. Safe supply concept document. 2019.

[39] Davidson PJ, Lopez AM, Kral AH (2018). Using drugs in un/safe spaces: impact of perceived illegality on an underground supervised injecting facility in the United States. Int J Drug Policy.

[40] Karazivan P, Dumez V, Flora L, Pomey MP, Del Grande C, Ghadiri DP (2015). The patient-as-partner approach in health care: a conceptual framework for a necessary transition. Acad Med.

[41] Strike C, Millson M, Hopkins S, Smith C (2013). What is low threshold methadone maintenance treatment?. Int J Drug Policy.

